# Atrial Fibrillation in Heart Failure: Novel Insights, Challenges, and Treatment Opportunities

**DOI:** 10.1007/s11897-024-00691-9

**Published:** 2024-11-22

**Authors:** Ghassan Bidaoui, Ala’ Assaf, Nassir Marrouche

**Affiliations:** Tulane Research Innovation for Arrhythmia Discovery (TRIAD), New Orleans, LA USA

**Keywords:** Atrial myopathy, Heart failure, Atrial fibrillation, Artificial intelligence, Catheter ablation

## Abstract

**Purpose of Review:**

Atrial fibrillation and heart failure frequently co-exist. This review discusses the comorbidity of atrial fibrillation and heart failure, the bi-directional link between them, and the recent advances in the management of these co-existing diseases.

**Recent Findings:**

Catheter ablation received a class 1 A recommendation for patients with AF and HF, after overwhelming evidence in heart failure with reduced ejection fraction and end-stage heart failure, while clinical trials are still lacking in patients with preserved ejection. Guideline-medical therapy of heart failure decreases the incidence of atrial fibrillation and the progression of atrial myopathy.

**Summary:**

Based on the current evidence, management of patients with both HF and AF should be include early optimization of comorbidity control, guideline-medical therapy for heart failure, and rhythm control preferentially through catheter ablation in properly selected patients.

## Introduction

Atrial fibrillation (AF) and heart failure (HF) are two prevalent cardiovascular diseases that frequently co-exist and lead to substantial morbidity and healthcare burden [1, 2]. Both diseases share the same risk factors and have an inter-related bidirectional pathophysiology. AF can be the cause or the consequence of HF. Regardless, when AF is diagnosed in a patient with HF, it has a prognostic impact on these patients, leading to more hospitalizations, strokes, and mortality. Inversely, HF can lead to the development and maintenance of AF, carrying with it the same dire consequences. The management of both diseases has witnessed significant improvements and paradigm shifts in the last decade. However, multiple gaps and difficulties are still faced in the management of these patients. In this review, we present the current understanding of the common pathophysiology between those diseases, the current state of management of these diseases, and the possible gaps that need to be filled in the future.

### Epidemiology and Prognostic Implications

AF is the common arrhythmia worldwide with approximately 33.5 million affected [1]. AF is diagnosed in more than half of the patients with new-onset HF, 44% of patients with acute HF, and 33% of patients with chronic HF [3]. Moreover, the prevalence of AF increases with increasing severity of HF. The implications of AF incidence in patients with HF are significant. AF is associated with worsened quality of life (QoL), refractory symptoms, increased hospitalizations, and mortality in HF patients. The increased risk in these patients has been shown to be found in all subtypes of HF regardless of ejection fraction (EF). Moreover, the AF burden and persistence has been shown to be strongly correlated with these outcomes, including hospitalizations and mortality.

Similarly, the incidence and prevalence of HF increases with increased AF severity, ranging from 33% in paroxysmal AF to 56% in permanent AF [4]. Although stroke is the most discussed complication of AF, HF is the most witnessed complication and is the main cause of hospitalization and mortality in these patients [5]. Furthermore, HF with preserved ejection fraction (HFpEF) has been shown to be more common in patients with AF and is associated with the same negative prognostic impact [6].

### Atrial Fibrillation Leading to Heart Failure

**Fig. 1:**
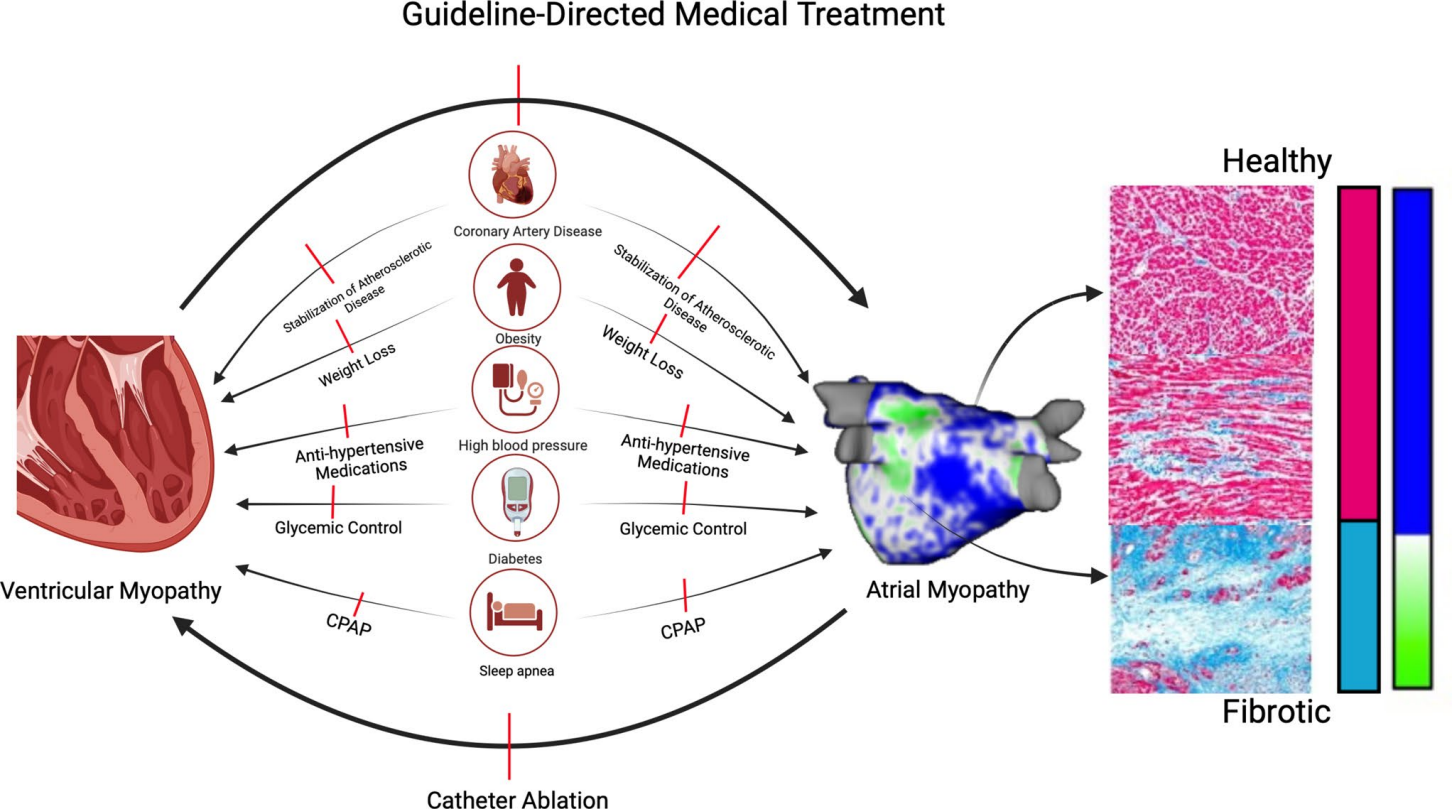
The Two Myopathies and the link between them. Legend: Early aggressive management of comorbidities, GDMT treatment for heart failure, and catheter ablation for atrial fibrillation should be combined to stop the vicious cycle. Created with Biorender

The propensity of AF and HF to co-exist may be caused by the shared risk factor profile of both disease and the fact that either of these diseases can lead to the other (Fig. 1) [7]. For example, aging pathways have been shown to be associated with increased reactive oxygen species, chronic inflammation, increased cellular senescence, and apoptosis of myocardial cells, both in the atrium and the ventricles, leading to the development of either AF through atrial remodeling or heart failure [8].

AF leads to the development and worsening of HF through multiple mechanisms including tachycardia-induced cardiomyopathy [9], irregular heart rate [10], loss of atrial kick [11], functional mitral regurgitation [12], and RAAS activation. First, Increased baseline heart rate and fast ventricular heart rate have been shown to lead to left ventricular impairment through increasing left ventricular pressure, abnormalities in calcium loading in the sarcoplasmic reticulum, and decreased diastolic filling time, leading to decreased cardiac output, activation of the renin-angiotensin-aldosterone-system (RAAS), and the eventual onset of adverse remodeling such as apoptosis, necrosis, and fibrosis development [9]. Second, irregular heart rate has been shown to lead to a significant reduction in cardiac output up to 9% [10]. Third, one of the functions of the left atrium is atrial systole, in which the atrium contributes to 25% of ventricular filling [11]. With fibrillatory rhythm, the atrial contribution to ventricular filling is blunted, leading to ineffective ventricular output. Finally, atrial myopathy, the complex structural, architectural, functional, and electrophysiological remodeling underlying AF is often correlated with left atrial dilation. Atrial dilation can lead to functional mitral regurgitation, increasing left ventricular filling pressures, ultimately leading to adverse ventricular remodeling [12].

### Heart Failure Leading to Atrial Fibrillation

HF also begets AF, albeit through both similar and different pathways. Both systolic and diastolic dysfunction in the left ventricle led to increased left ventricular and atrial filling pressures [13]. Increased atrial pressure is associated with increased wall stress. Increased wall stress will activate multiple remodeling pathways including neurohormonal, pro-fibrotic and pro-inflammatory pathways, ultimately leading to the development of atrial myopathy. The RAAS system promotes atrial remodeling through the increased expression and activation of multiple pro-fibrotic and pro-inflammatory molecules including but not limited to the mitogen-activated protein kinase (MAPK), the Janus kinase (JAK)/signal transducers and activators of transcription (STAT), transforming growth factor β1 (TGF-β1), angiotensin II activated platelet-derived growth factor A (PDGF-A), and nuclear factor-kappa B (NF-κB) [14]. Furthermore, autonomic dysregulation and sympathetic activation secondary to decreased cardiac output have been shown to lead to increase AF inducibility and trigger firing.

**Fig. 2:**
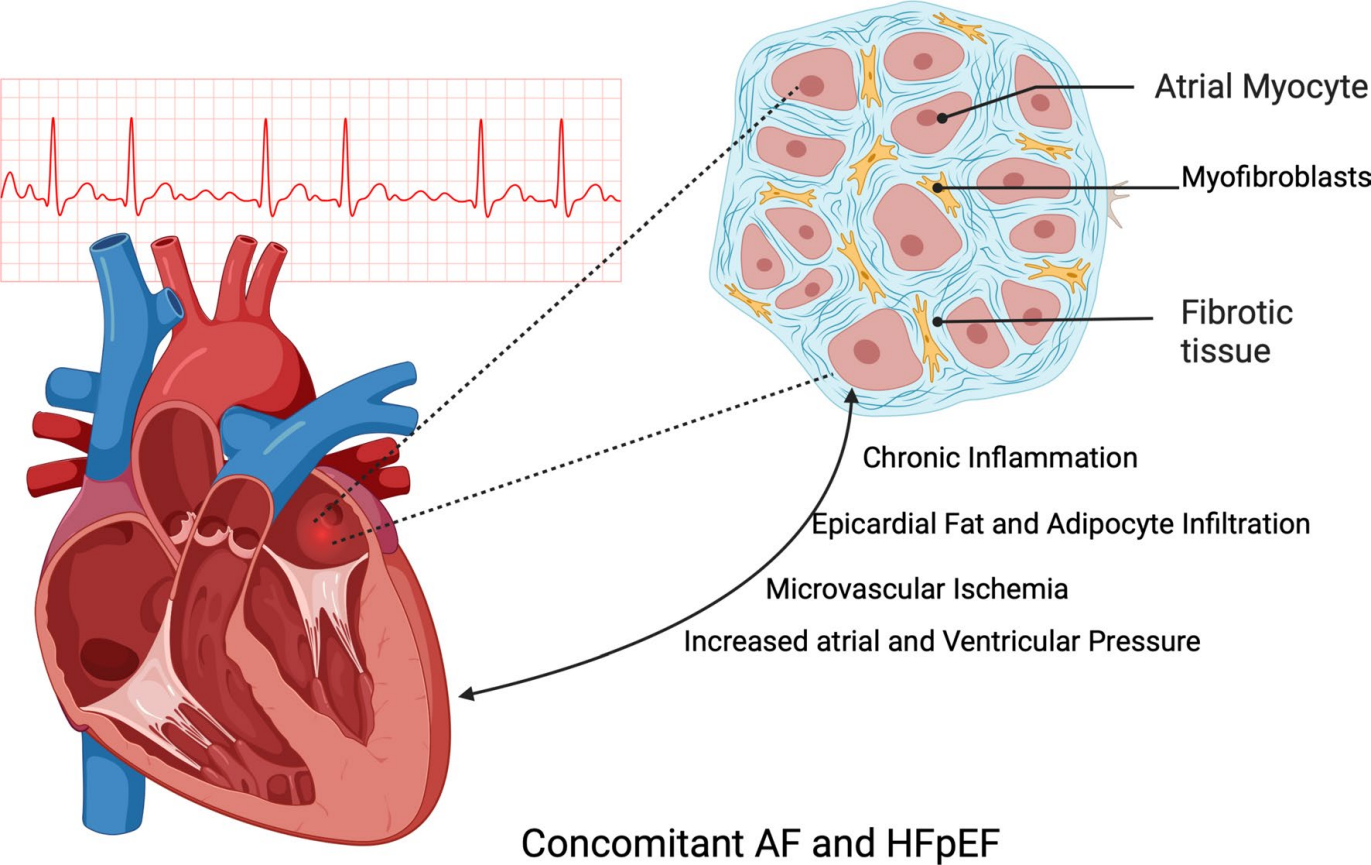
The bi-directional relationship of atrial myopathy and HFpEF. Legend: HFpEF and atrial myopathy are linked by shared mechanisms: chronic inflammation prompting myocardial remodeling, epicardial fat contributing to structural changes, microvascular ischemia influencing both conditions, and diastolic dysfunction elevating cardiac pressures. Created with Biorender

The pathophysiology of atrial myopathy differs between HF with reduced ejection fraction (HfrEF) and HF with preserved ejection fraction (HFpEF), however. In HFpef, left atrial remodeling is only partially driven by increasing pressures secondary to decreased compliance and atrioventricular coupling. The primary driver is shown to be chronic systemic inflammation that leads to the remodeling of both the ventricles and the atria concurrently **(**Fig. 2) [15, 16]. For example, obesity and increased pericardial fat have been shown to be implicated in both the development of HFpeF and atrial myopathy through the release of paracrine inflammatory and pro-fibrotic molecules and the infiltration of adipocytes to the myocardium [17, 18]. Increased epicardial fat has been shown to increase the incidence of AF and predict a worse trajectory in patients with established AF [19].

### Targeting Ventricular Myopathy: Treat the Heart Failure

Given the incremental yield of ventricular dysfunction on the development and progression of atrial myopathy, early and dynamic optimization of guideline-directed medical therapy (GDMT) is crucial as GDMT components have been shown to improve AF burden in AF HF patients [20]. Angiotensin Receptor-Neprilysin Inhibitor (ARNI) decreases morbidity and mortality in HFrEF patients and has beneficial effects on HFpEF and HF with mid-range ejection fraction (HFmrEF) patients, and especially in patients with lower EF [21]. Although ARNIs were not shown to decrease AF incidence compared to Angiotensin-Converting Enzyme (ACE) inhibitors or Angiotensin II Receptor Blocker (ARB)s in a meta-analysis and in a PARAGON-HF (Prospective Comparison of ARNI with ARB Global Outcomes in HF with Preserved Ejection Fraction) post-hoc analysis, it was associated with favorable atrial remodeling after CA compared to valsartan [22, 23]. AF status did not affect the benefit attained from the use of ARNI in HFrEF patients both in the PARADIGM-HF (Prospective comparison of ARNi with ACEi to Determine Impact on Global Mortality and morbidity in Heart Failure and PARAGON-HF studies [24]. Hence, all HFrEF patients with AF should be on ARNI, with selective administration in patients with HFpEF or HFmrEF.

In patients with contraindications or inability to achieve ideal ARNI use, ACE inhibitors or ARBs should be given for HFrEF given their favorable effect on hospitalization and mortality [25]. Both trandolapril and enalapril in a sub analysis decreased the incidence of new-onset AF in HFrEF patients [26, 27]. ACE inhibitors also decreased the severity of AF recurrences as signified by decreased AF-related hospitalizations and mortality [28].

The same benefits can be achieved with ARBs. In the Val-HeFT (Valsartan Heart Failure Trial) and the CHARM (Candesartan in Heart failure: Assessment of Reduction in Mortality and morbidity) trial, patients receiving ARBs arm developed less incident AF compared to patients on placebo [29, 30]. Although no studies have investigated the effect of developed AF on the benefits of ACE or ARBs in these patients, an analysis from the CHARM trial showed that candesartan retained its beneficial effects on mortality and HF-related hospitalization regardless of baseline AF status [29, 30].

Aldosterone is activated through the RAAS pathway and has been shown to increase local conduction times, p-wave duration, and right atrial activation time in rats on electrophysiologic mapping. Furthermore, it has been shown to be associated with increased atrial fibrosis and fibroblast proliferation [31]. Hence, MRAs, by inhibiting aldosterone were shown to decrease both atrial fibrosis and AF development [32]. In a sub analysis of the EMPHASIS-HF (Eplerenone in Mild Patients Hospitalization and Survival Study in Heart Failure) trial, eplerenone decreased the incidence of new-onset AF. It also decreased cardiovascular deaths or hospitalizations regardless of baseline AF [33]. However, spironolactone did not exhibit the same effect on incident AF in HFpEF patients in the TOPCAT (Treatment of Preserved Cardiac Function Heart Failure with an Aldosterone Antagonist) and IMPRESS-AF (Improved Exercise Tolerance in Heart Failure with Preserved Ejection Fraction by Spironolactone on Myocardial Fibrosis in Atrial Fibrillation) trials [34, 35]. Hence, Mineralocorticoid Receptor Antagonist (MRA)s should be administered for HFrEF to improve outcomes, with selective administration in HFpEF patients for symptomatic relief.

Sodium-Glucose Co-transporter-2 (SGLT-2) inhibitors are the only medications that have shown prognostic efficacy in all patients with heart failure regardless of diabatic status, or EF pertaining to HF hospitalizations and cardiovascular mortality [6, 36]. In most of the studies including DAPA-HF (Dapagliflozin and Prevention of Adverse-outcomes in Heart Failure), EMPEROR-Preserved (Empagliflozin outcome trial in patients with chronic HF with Preserved Ejection Fraction) and DELIVER (Dapagliflozin Evaluation to Improve the Lives of Patients with Preserved Ejection Fraction Heart Failure), SGLT-2 use was not associated with decreased AF incidence [6, 37, 38]. However, in DECLARE-TIMI 58, dapagliflozin did decrease Atrial fibrillation and atrial flutter incidence regardless of heart failure presence at baseline. Importantly, atrial fibrillation diagnosis at baseline did not affect the positive effect of these medications on both hard and soft outcomes in these studies [39].

## Catheter Ablation: The Fifth Guideline-Directed Therapy

### Rhythm or Rate Control

Given the progressive and self-perpetuating nature of atrial myopathy and its effect on ventricular function, rhythm control is expected to be associated with improved outcomes in AF HF patients. Previously, rate control was favored as the first-line treatment based on key trials comparing rhythm and rate control. These trials, mainly the AFFIRM (Atrial Fibrillation Follow-up Investigation of Rhythm Management, RACE (Rate Control versus Electrical Cardioversion for Persistent Atrial Fibrillation), and STAF (Strategies of Treatment of Atrial Fibrillation) trials demonstrated no significant benefit from rhythm control, despite the expected better sinus rhythm management [40–42]. Moreover, signals of harm with rhythm control were demonstrated in these studies with amiodarone being associated with increased hospitalizations, bradycardia, and mortality. For example, The AF CHF (Atrial Fibrillation and Congestive Heart Failure) trial enrolled 1376 AF HF with an EF of less than 35% and compared stroke, decompensated HF events, and mortality in patients on rhythm control (82% amiodarone) or rate control [43]. No significant difference was shown between rhythm and rate control for these outcomes after 4 years of follow-up (hazard ratio in the rhythm-control group, 1.06; 95% confidence interval, 0.86 to 1.30; *P* = 0.59). These outcomes were further echoed in earlier meta-analysis of these trials favoring rate-control.

However, multiple caveats should be kept in mind when understanding the mentioned trials, multiple caveats should be kept in mind. First, rhythm control was done through amiodarone administration, a drug known to be associated with multiple cardiac and non-cardiac toxicities. It has been shown repeatedly that amiodarone leads to more hospitalizations and all-cause mortality. These adverse outcomes might have negated the beneficial effects of sinus rhythm. Second, in that era, the management of the comorbidities that lead to increased events including HF was not as optimal as now, and the antiarrhythmics dosing might have been inappropriate, leading to reduced effect or increased ventricular and atrial arrhythmogenic potential.

Recent clinical trials have shown a merit to rhythm control, especially through CA, in HF patients. The ARC-HF (A Randomized Trial to Assess Catheter Ablation Versus Rate Control in the Management of Persistent Atrial Fibrillation in Heart Failure) in 2013 assessed the impact of CA compared to rate control on peak oxygen consumption after one year of follow-up [44]. The trial enrolled 52 patients with HFref with an EF of less than 35% and persistent atrial fibrillation. CA led to significantly more improvement in peak oxygen consumption compared to the rate control arm (difference + 3.07 ml/kg/min, 95% confidence interval: 0.56 to 5.59, *p* = 0.018)). Moreover, CA was significantly better in maintaining sinus rhythm and led to improvements in QoL (*p* = 0.019) and B-type Natriuretic Peptide (BNP) (*p* = 0.045). The CAMTAF (Catheter Ablation for the Management of Atrial Fibrillation in Patients with Heart Failure), a one-center unblinded study, enrolled 50 patients with HFrEF and EF < 50%, 56% of whom had a co-diagnosis of HF and AF at the same time, suggesting tachycardia cardiomyopathy. In this study, CA was shown to improve peak VO2 (22 ± 6 versus 18 ± 6 mL/kg per minute; *P* = 0.014), EF, (40 ± 12% compared with 31 ± 13%; *P* = 0.015) and QoL (24 ± 22 versus 47 ± 22; *P* = 0.001). The CAMERA-MRI (Catheter Ablation versus Medical Rate Control in Atrial Fibrillation and Systolic Dysfunction) trial included 68 patients with nonischemic cardiomyopathy and AF. The study showed that EF (18% vs. 4.4; *p* < 0.001), and New York Heart Association (NYHA) class improved in the CA arm(*p* = 0.001) [45].

To eliminate the possibility that insufficient rate control was the main driver of these positive findings, the PABA-CHF (Pulmonary Vein Antrum Isolation versus AV Node Ablation with Biventricular Pacing for Treatment of Atrial Fibrillation in Patients with Congestive Heart Failure) assessed the effectiveness of CA compared to the most extreme rate control strategy, AVN + CRT [46]. The study included 81 patients with an EF < 40% and most of the patients had drug-resistant AF with mean duration of AF of 4 years. The study showed that CA improved functional and hemodynamics such as EF (35% vs. 28%, *p* < 0.001), Qol (60 vs. 82; *p* < 0.001), and Six-Minute Walk Distance (6MWD) test (340 vs. 297 m, *p* < 0.001) more compared to rate control. CA was better at maintaining sinus rhythm. These small trials comparing CA and rate control consistently demonstrated the superiority of rhythm control through CA compared to all measures of rate control in AF HF patients.

Then, multiple studies have also compared CA with pharmacological medical therapy in AF HFreF patients as pertaining to hard outcomes. The largest study to compare CA with pharmacological management of AF in HFrEF patients is the CASTLE-AF (Catheter Ablation versus Standard Conventional Treatment in Patients with Left Ventricular Dysfunction and Atrial Fibrillation) trial [47]. In the CASTLE-AF (2018) trial, 3013 patients were assessed for inclusion and 398 were enrolled at 33 sites in 3 continents from January 2008 to January 2016. Patients included were patients with symptomatic paroxysmal or persistent AF who were refractive to pharmacological rhythm control, had side effects, or were unwilling to take medications. The patients had an LVEF of less than 35% and an ICD implanted. Both groups were treated with GDMTs with most on beta blockers, ACE or ARB, and diuretics including spironolactone. Operators were advised to ablate until ablation is successful with additional substrate modification as needed. The primary endpoint of the study was the composite of all-cause mortality and hospitalization for worsening heart failure. Catheter ablation demonstrated superiority compared to medical therapy pertaining to the primary endpoint after a median follow-up of 3 years (28.5% vs. 44.6%, *p* = 0.007). This was driven by lower all-cause mortality (24 [13.4%] vs. 46 [25.0%]; HR, 0.53; 95% CI 0.32 to 0.86; *p* = 0.01) and hospitalizations, whether related to worsening heart failure (37 [20.7%] vs. 66 [35.9%]; HR 0.56; 95% CI.

0.37 to 0.83; *p* = 0.004) or cardiovascular disease (111.2% vs. 22.3%; *p* = 0.009). Moreover, LVEF improvement was higher in patients who under CA compared to medical therapy. The superiority of CA compared to pharmacological therapy was also shown in multiple other studies including the CAPTAF (Catheter Ablation for Paroxysmal Atrial Fibrillation) and the AATAC (Ablation vs. Amiodarone for Treatment of Atrial Fibrillation in Patients with Congestive Heart Failure and an Implanted ICD/CRTD) trial [48, 49].

### The Earlier the Better

Blunting or slowing down the progression of atrial myopathy secondary to continuous fibrillatory rhythm is expected to improve outcomes in AF HF patients and decrease the risk of stroke. The EAST AFNET4 (Early Treatment of Atrial Fibrillation for Stroke Prevention Trial) trial enrolled patients with AF diagnosed within the last 12 months before randomization [50]. The patients were randomized to either usual care or usual care plus early rhythm control treatment. The primary outcome was a composite of cardiovascular-related deaths, stroke, or hospitalization associated with worsening HF or acute coronary syndrome. 20% of the patients in the rhythm control underwent CA as the early rhythm control method. Early rhythm control was associated with decreased risk of the primary composite outcome with no significant difference in the primary safety outcome (Hazard ratio = 0.79, *p* = 0.005).

A sub-analysis of the EAST AFNET4 trial was done focusing on HF patients (mostly HFpEF, *n* = 442) proving that early rhythm control should be pursued in AF HF patients(HR = 0.74 *p* = 0.03) [51]. Another proof to the merit of early rhythm control for AF HF patients emerged from sub analysis from the CASTLE-AF and CABANA (Catheter Ablation vs. Anti-arrhythmic Drug Therapy for Atrial Fibrillation) trials [52, 53]. In Castle-AF, multiple sub analysis has shown that response to CA was heterogeneous. Patients with more severe HF, that is NYHA score greater or equal to 2, ischemic cardiomyopathy, or an EF less than 25% did not benefit as much. These results show that early CA before the advanced stages of HF is reached can improve the outcomes of these patients. Corroborating these results, a sub analysis from CAMERA-MRI showed that EF improved the most in patients with no ventricular fibrosis on LGE-MRI. Finally, in the CABANA trial, patients who are younger and/or have paroxysmal AF had better outcomes compared to older and/or persistent AF patients.

### Never Too Late to Ablate

What about patients with end-stage disease or advanced myopathy? Does rate control the solution? CASTLE-HTX compared catheter ablation with medical therapy alone in these patients [54]. This study was a single-center randomized trial done in Germany that enrolled 194 patients with symptomatic AF and end-stage HF eligible for heart transplant for the primary endpoint of the composite of all-cause mortality, implantation of a left ventricular assist device or urgent heart transplantation. All patients had to have an implantable cardiac device with activated arrhythmia detection to allow for continuous monitoring in the follow-up for arrhythmia recurrence. The trial was stopped early for efficacy; with a significant reduction in the primary endpoint in the CA arm after a median follow-up of 18 months (HR = 0.24, *p* < 0.001). Also, no significant difference in complications was seen between the two arms. Interestingly, CA also appeared to modulate the progression of AF, with more patients reverting to paroxysmal AF from persistent AF in the ablation arm (71–36%) compared to the medical therapy arm (68–61%).

A sub analysis investigated the predictors of outcomes in patients with end-stage HF and showed that an EF of less than 30%, NYHA class equal or greater than 3 and an increased AF burden was associated with primary outcomes. Then, based on these three factors, patients were divided into high-risk and low-risk groups. Interestingly, the patients who reaped the most benefit from CA were the patients who were at high-risk for outcomes(IR = 24.69 vs. 3.7) [55].

### Catheter Ablation in Heart Failure with Preserved Ejection Fraction

Clinical trial data on CA efficacy in patients with HFpEF is not as robust as for those with HFrEF, being mostly limited to single-center trials, sub-studies of larger trials, or registry-based analyses. Nevertheless, emerging data points to the potential benefits of catheter ablation in this patient population. A sub-analysis of the EAST AFNET4 trial, which included around half of the patients with HFpEF out of a total of 798 HF patients, suggested that early rhythm control confers benefits in HFpEF patients [51]. These benefits included improved NYHA class and a lower risk for primary composite outcomes. Parallel insights emerge from a sub-analysis of the CABANA trial, where most of the HF subset had HFpEF [56]. Results indicated a substantial relative reduction in a composite endpoint with CA compared to medical therapy (hazard ratio, 0.64 [95% CI, 0.41–0.99]).

Smaller studies also support the potential advantages of ablation for HFpEF patients, showing improvements in parameters like peak exercise pulmonary capillary wedge pressure (PCWP) ((30.4 to 25.4 mm Hg; *P* < 0.01)), peak relative VO2((20.2 to 23.1mL/kg/min; *P* < 0.01)), NT-proBNP((794 to 141 ng/L; *P* = 0.04)), and QoL metrics(51 to 16.6; *P* < 0.01) [57]. Additionally, these studies indicated that a significant proportion of patients might no longer meet the criteria for HFpEF after ablation (50% vs. 7%, *p* < 0.01). In the context of Japanese multicenter registry data, CA was associated with a significantly reduced risk of cardiovascular death and HF hospitalization compared to medical therapy after an average follow-up of about two years (HR = 0.32, *P* = 0.004) [58]. Complementing this, a meta-analysis of seven observational studies found no significant difference in recurrence or stroke when looking specifically at HFpEF status, and that HFpEF and HFrEF patients appeared to have comparable outcomes(*p* = 0.59) [59]. These findings collectively indicate a growing body of evidence suggesting that CA may offer benefits to patients with HFpEF, mirroring some of the positive outcomes seen in patients with HFrEF. However, the literature is still lacking, and randomized clinical trials are needed.

### Preventing Stroke

AF, to a larger extent, and HF are prothrombotic states, and the co-existence of both is associated with an increased risk for debilitating strokes. AF-related stroke is more severe, function-limiting, and recurring compared to non-AF stroke and accounts to one-third of all thromboembolic events. The increased risk of stroke in AF patients is multifaceted. A rough endocardium as described by Masawa et al. in the left atrium secondary to atrial fibrosis and adverse atrial remodeling forms a prothrombotic and pro-inflammatory substrate for thrombus formation [60]. Atrial fibrosis, as detected on LGE-MRI, is a strong predictor of thromboembolic events [61]. This association has been demonstrating even in patients with no AF [62]. The irregular rhythm and the dilated left atrium also promote stroke through blood stasis. Increased platelet activation and inflammatory markers such as C-Reactive Protein (CRP), interleukins, and Von Willebrand Factor (VWF) in AF patients have been shown to be associated with stroke.

Kang et al. reported that patients with concomitant AF and HF have a five-fold higher risk of stroke compared to patients with neither [63]. The increased risk of stroke in these patients can be seen both in patients with HFrEF and HFpEF [64]. The management of thrombotic risk is paramount in patients with both diseases. Stroke risk assessment and patient selection for anticoagulation is based on CHA2DS2-VASc score, which incorporates HF as a factor (C). However, in patients with both conditions, the discriminatory power of CHA2DS2-VASc decreases, limiting its utility. Also, it does not incorporate the severity, subtype, and duration of heart failure, all shown to be determining factors of stroke. Hence, a much lower threshold for anticoagulation should be utilized in patients with both diseases. The anticoagulation strategy does not differ from the one recommended for AF only patients, with Direct Oral Anticoagulants (DOACs) as the most preferred option. DOACs incur a lower risk of bleeding and a more lenient requirement for follow-up. Given the older age, polypharmacy, and multi-comorbidity expected in AF + HF patients, these benefits are particularly relevant. Warfarin is only to be used in patients with end-stage chronic kidney disease and mitral valve disease. Antiplatelet therapy on top of anticoagulation, especially in HF patients, is associated with an intolerable risk of bleeding. Hence, antiplatelet should be avoided and even stopped in the setting of stable atherosclerotic disease. To sum up, anticoagulation is preferred in all patients with AF + HF in the right clinical context.

### Future Directions and Gaps

Although significant progress has been made, there are still many gaps in knowledge and opportunities for improvement. First and foremost, while the connection between atrial myopathy, atrial fibrillation (AF), and heart failure (HF) has been established, a comprehensive understanding of atrial myopathy—including its varied structural, electrophysiological, and mechanical remodeling across individuals—remains incomplete. Additionally, the optimal management of atrial myopathy prior to the onset of AF is unclear. Given that atrial myopathy without AF has been associated with an increased stroke risk, the question arises: should we administer anticoagulation if severe atrial enlargement or advanced fibrosis is detected on Late Gadolinium LGE-MRI in patients with heart failure? Whether the CHA2DS2-VASc score should be applied in these cases is also unknown [65, 66].

**Fig. 3:**
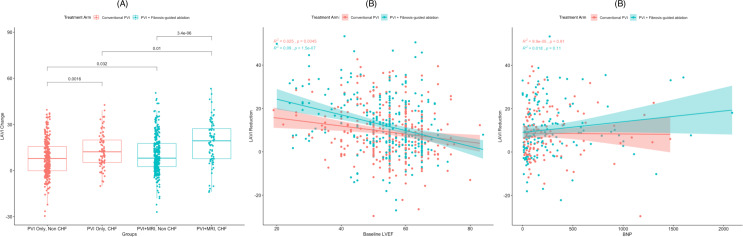
Left Atrial volume index reduction in heart failure patients with substrate modification. Legend: Left atrial volume index was reduced in HF patients after catheter ablation in the DECAAF II trial. The favorable remodeling was most noticeable in in HF patients who underwent substrate modification

To date, pulmonary vein isolation remains the primary catheter ablation technique with demonstrated efficacy in the general population, while substrate modification is typically reserved for select cases based on physician preference [67]. The specifics of which type of substrate modification should be performed, its extent, and the patient profiles that are most likely to respond favorably are still to be determined, especially in patients with comorbidities such as HF. In a DECAAF II sub analysis, we have shown that CA with substrate modification led to more favorable remodeling indicated by decreased LAVI compared to pulmonary vein isolation alone. Interestingly, patients who had the most remodeling in DECAAF II with substrate modification were patients with heart failure, regardless of ejection fraction. Moreover, we found in our patients, substrate modification through MRI-guided ablation led to better results compared to pulmonary vein isolation in younger patients compared to older population, a finding that echoes the findings of a CABANA sub analysis showing that younger people in general respond better to ablation (Fig. 3) [52, 68]. Given the retrospective nature of these analysis, they serve as a hypothesis-generating. However, it clearly shows that aging, the strongest risk factor for atrial fibrillation, modulates atrial myopathy albeit in ways we do not know clearly. Given the strong association of age with most cardiovascular diseases including heart failure, investigating aging pathways including senescent pathways specifically related to atrial and ventricular myopathy can lead to the identification of common therapeutic targets.

Lastly, stroke prediction continues to rely on the CHA2DS2-VASc score, neglecting AF burden and persistence, heart failure subtypes, imaging indices, genetic factors, or the severity, duration, or treatment of comorbidities in stroke risk assessment [3]. Additionally, the heterogeneous nature of patients with both AF and HF calls for a phenotyping approach that could facilitate more personalized and targeted management strategies [69, 70]. Till now, guidelines and current medical practice gives recommendation to follow for AF and HF in general given the lack of established phenotypes that precisely characterizes the heterogeneity of both diseases and the interactions between them.

**Fig. 4:**
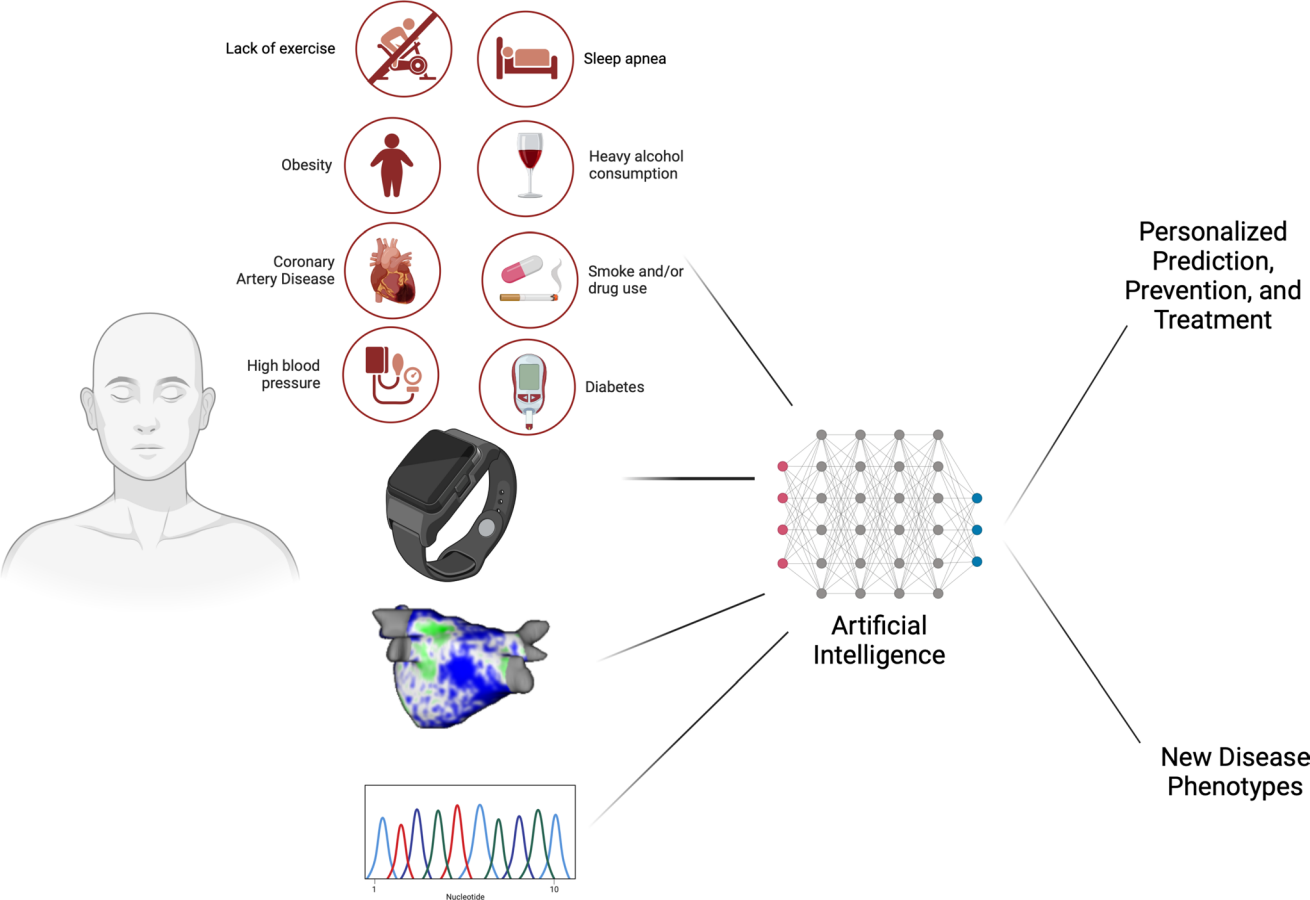
The future of personalized medicine. Legend: This diagram illustrates the integration of multimodal data to tailor AF and HF management. Comorbidities, biometrics from smartwatches, atrial myopathy imaging, and genomics will be integrated. These data are then analyzed by artificial intelligence algorithms to enhance patient-specific disease trajectory predictions, prevention strategies, and treatment plans, potentially leading to the identification of novel disease phenotypes depicting the heterogeneity of both diseases. Created with Biorender

**Fig. 5:**
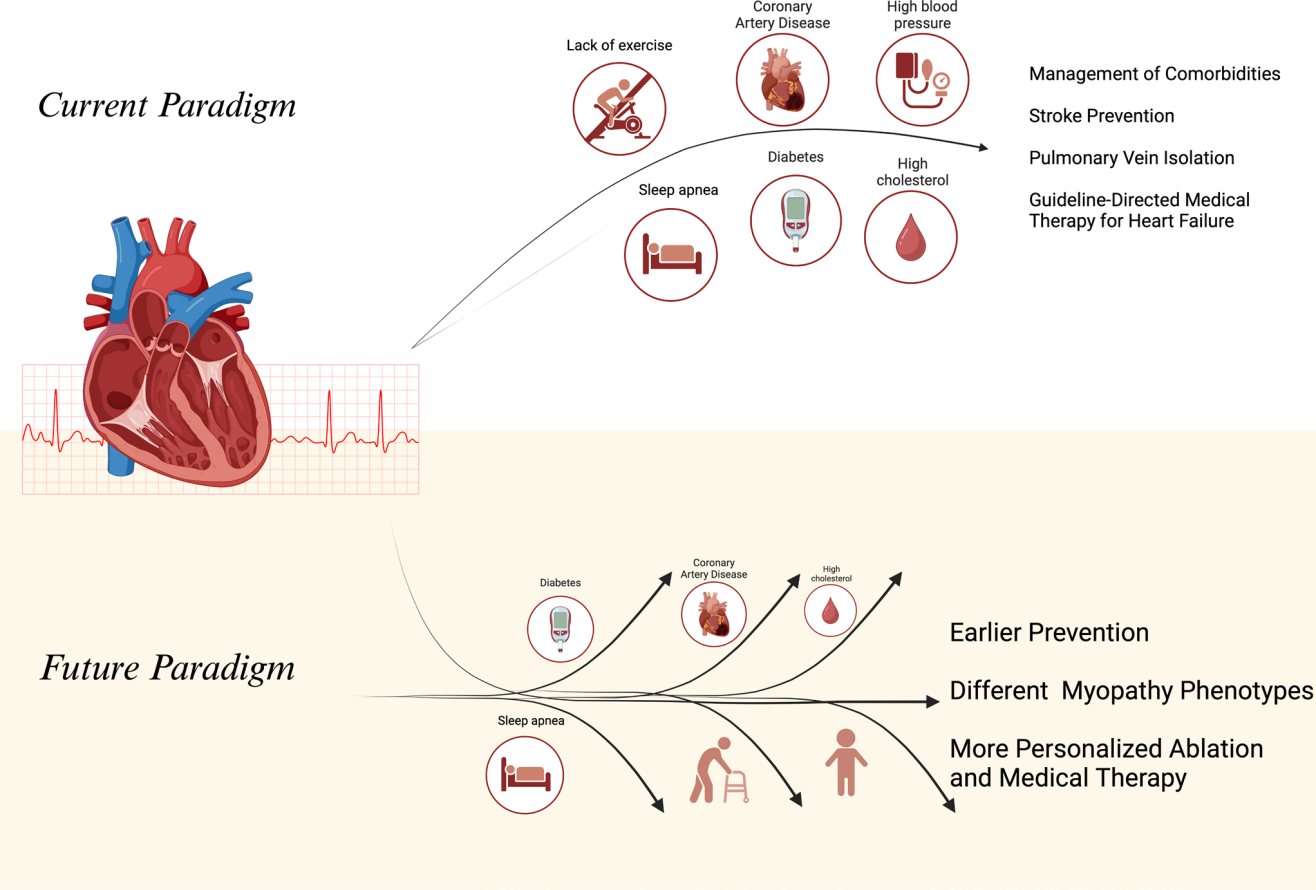
Future Directions in management. Created with Biorender

To personalize the treatment of concomitant AF and HF, it is imperative to harness big data from wearable technology, atrial and ventricular myopathy imaging, advanced genomic profiling, and the comprehensive analysis of patient comorbidities and biomarkers (Fig. 4) [71]. The future of patient-centric care lies in the strategic application of artificial intelligence (AI), including machine learning and deep neural networks, to dissect vast databases. By doing so, we can significantly refine the phenotyping of AF and HF patients. The implementation of AI could facilitate the identification of subtle patterns and predictors of disease progression and response to treatment that remain elusive to conventional analysis. This approach could lead to the improved prediction, prevention, and treatment of both AF, HF, and AF-HF (Fig. 5).

## Conclusion

Early is key in the management of patients with comorbid AF and HF. Targeting atrial myopathy, through early rhythm control, aggressive comorbidity control, proper anticoagulation, and guideline-directed medical therapy prove effective. While catheter ablation is beneficial for those with reduced ejection or end-stage HF, its utility in HFpEF requires further clarity. Future directions must aim to deepen our understanding of atrial myopathy and its progression, and better define patient phenotypes to personalize treatment effectively.

## Data Availability

No datasets were generated or analysed during the current study.

## Abbreviations

AF: Atrial Fibrillation
HF: Heart Failure
NYHA: New York Heart Association (used for classifying the extent of heart failure)
EF: Ejection Fraction
RAAS: Renin-Angiotensin-Aldosterone System
MAPK: Mitogen-Activated Protein Kinase
JAK/STAT: Janus Kinase/Signal Transducers and Activators of Transcription
TGF-β1: Transforming Growth Factor Beta 1
PDGF-A: Platelet-Derived Growth Factor A
NF-κB: Nuclear Factor kappa-light-chain-enhancer of activated B cells
GDMT: Guideline-Directed Medical Therapy
ARNI: Angiotensin Receptor-Neprilysin Inhibitor
ACE: Angiotensin-Converting Enzyme
ARB: Angiotensin II Receptor Blocker
MRA: Mineralocorticoid Receptor Antagonist
SGLT-2: Sodium-Glucose Co-transporter-2
PCWP: Pulmonary Capillary Wedge Pressure
VO2: Volume of Oxygen
NT-proBNP: N-Terminal pro B-type Natriuretic Peptide
LGE-MRI: Late Gadolinium Enhancement Magnetic Resonance Imaging
CRT: Cardiac Resynchronization Therapy
6MWD: Six-Minute Walk Distance
HFpEF: Heart Failure with Preserved Ejection Fraction
HFrEF: Heart Failure with Reduced Ejection Fraction
HFmrEF: Heart Failure with Mid-Range Ejection Fraction
DOACs: Direct Oral Anticoagulants
CRP: C-Reactive Protein
VWF: Von Willebrand Factor
QoL: Quality of Life
